# Slowing Myopia Progression in Adolescents Using Orthokeratology: A Retrospective Chart Review

**DOI:** 10.7759/cureus.87073

**Published:** 2025-06-30

**Authors:** Bradley A Nordin

**Affiliations:** 1 Ophthalmology, Huffman and Huffman, PSC, London, USA

**Keywords:** adolescents, appalachia, corneal curvature, myopia progression, ortho-k, orthokeratology, refractive error, rural eye care

## Abstract

Introduction: This study evaluated the effectiveness of orthokeratology (ortho-k) in reducing myopia progression rates in adolescents from a rural Appalachian community by comparing pre- and post-treatment clinical measures of refractive error and corneal curvature.

Methods: A retrospective chart review was conducted on 45 adolescents (90 eyes, aged 8-16) who had been treated with ortho-k for at least six months at a rural optometry practice. Post-treatment clinical data were obtained between 21 and 90 days of treatment discontinuation. Refractive error, corneal curvature (k values), and adverse events were collected and statistically analyzed, with myopia progression rates compared to the published untreated rate of -0.50 diopters per year (D/y).

Results: The mean myopia progression rate was -0.227 ± 0.351 D/y, significantly slower than the untreated rate (p < 0.001). A subgroup with corneal flattening (n = 11 eyes) showed a progression rate of -0.028 ± 0.134 D/y. Post-treatment corneal curvature steepened on average (Δkm = 0.327 ± 0.445 D), but flattening was linked to slower progression. No significant effects of sex, age, or treatment duration were found in the full sample, although male patients in the flattened subgroup had slower progression than female patients (p = 0.014). No adverse events were reported.

Conclusion: Ortho-k significantly reduces myopia progression in adolescents, particularly in those with corneal flattening, as demonstrated by a slower progression rate compared to untreated individuals. Its safety and effectiveness make it a valuable treatment option for managing myopia in rural communities.

## Introduction

Myopia, a refractive error characterized by blurred distance vision due to excessive axial elongation of the eye, represents a significant global public health challenge. Projections estimate that by 2050, nearly five billion people (approximately half the world’s population) will be myopic, with up to one billion at risk of high myopia [1]. High myopia, defined as a spherical equivalent of -5.00 diopters (D) or worse, significantly increases the risk of vision-threatening complications, including retinal detachment, glaucoma, and myopic macular degeneration [2]. Adolescents are particularly susceptible to rapid myopia progression due to ongoing ocular growth, with untreated progression rates averaging -0.50 D per year (D/y) and up to -2.00 D/y in East Asian populations, where intensive near-work and limited outdoor time have led to as many as 90% of teenagers being myopic [3-6]. The COVID-19 pandemic has intensified this trend with a surge in screen exposure, accelerating myopia’s onset worldwide [7]. The rapid rise in myopia prevalence, particularly among children and adolescents, underscores the urgency of effective interventions [8,9]. This study defines adolescents as children aged 8-16, capturing the primary period of hormonal changes and growth associated with typical puberty in both boys and girls.

The progression of myopia is driven by excessive axial elongation, where biomechanical changes in the sclera stretch the retina, heightening the risk of vision-threatening conditions such as myopic macular degeneration and retinal detachment [8,10]. This process is particularly pronounced during adolescence, when ocular growth accelerates, making early intervention critical to mitigate long-term visual impairment [5]. Effective myopia control strategies aim to slow axial elongation, reducing the severity of refractive error and the associated socioeconomic burden of managing high myopia complications [11]. Given the rapid progression rates in untreated adolescents, interventions that target eye growth are crucial for preserving vision and enhancing quality of life.

Orthokeratology (ortho-k) is a non-surgical intervention that has gained attention for its dual benefits in correcting refractive errors and slowing the progression of myopia in adolescents. Ortho-k utilizes specially designed rigid gas-permeable contact lenses worn overnight to temporarily reshape the cornea, thereby correcting refractive errors such as myopia. The sustained application of gentle pressure to the corneal epithelium flattens or reduces the curvature of the central region, effectively decreasing the eye’s refractive power to shift the focal point onto the retina for clearer distance vision. In addition to flattening of the central cornea, ortho-k contacts also steepen the mid-peripheral cornea, creating a peripheral myopic defocus. This peripheral defocus is thought to inhibit axial elongation of the eye, a primary driver of myopia progression, by altering retinal signaling pathways that regulate eye growth, as demonstrated by both animal models and clinical studies [12,13]. The reshaping effect, however, is temporary and reversible, necessitating consistent nightly wear to sustain both the vision correction and the myopia control benefits. This unique optical profile positions ortho-k as a promising tool for managing myopia, particularly in young patients with progressive refractive errors.

Extensive research supports the efficacy of orthokeratology in slowing the progression of myopia. Randomized trials, such as the Retardation of Myopia in Orthokeratology (ROMIO) study, have demonstrated that ortho-k reduces axial length elongation by 30%-50% compared to spectacles or soft contact lenses [14], with similar findings in the Myopia Control Using Toric Orthokeratology (TO-SEE) study for patients with moderate astigmatism [15]. These results were further corroborated in a meta-analysis by Si et al., which found a pooled 45% reduction in progression [6]. Compared to low-dose atropine, which achieves a 30%-60% reduction but may cause photophobia, or multifocal soft contact lenses with 30%-40% efficacy that require daytime wear, ortho-k offers a non-invasive, lifestyle-compatible alternative with sustained benefits over long-term use [16]. Its overnight wear enhances vision-related quality of life, particularly for active adolescents, as supported by studies comparing contact lens modalities [17]. Additionally, ortho-k’s safety profile, with rare adverse events when proper hygiene is maintained, makes it suitable for diverse clinical settings [18].

Orthokeratology poses distinct economic and accessibility challenges in rural settings, such as the Appalachian community examined in this study. The high initial cost of custom-fitted lenses and follow-up care often exceeds that of traditional eyewear, creating a financial barrier for patients in economically disadvantaged regions with limited or no insurance coverage for specialty lenses. This challenge is compounded by the scarcity of providers offering ortho-k fittings, with most concentrated in urban areas. In the state of this study, only a small proportion of eye care providers offer ortho-k, primarily in metropolitan centers, resulting in increased travel time and expenses for rural patients [19]. Despite these obstacles, ortho-k offers significant value for rural Appalachian communities by reducing long-term healthcare costs and providing convenience. Its ability to correct vision without daytime eyewear minimizes the need for frequent prescription updates or lens replacements, easing the burden on patients with limited access to eye care [20]. Consequently, ortho-k represents both a clinically effective and practical solution for underserved populations, where economic constraints and geographic isolation often restrict access to advanced myopia management strategies [21]. While clinical trials provide robust evidence, real-world data from diverse populations are essential to confirm ortho-k’s practical efficacy. Rural settings, such as the Appalachian community in this study, provide a unique context in which lower exposure to digital devices and near-work may influence baseline progression rates [9,22].

The purpose of this study is to evaluate the effect of orthokeratology on slowing myopia progression in adolescents from a rural Appalachian community by measuring changes in refractive error and corneal curvature (k values) at least 21 days and within 90 days post-treatment discontinuation. The primary null hypothesis is that there is no difference in the rate of myopia progression (e.g., -0.50 D/y or greater) between adolescents treated with ortho-k and published rates for untreated myopic adolescents, indicating no effect on controlling axial elongation [3,23]. The alternative hypothesis posits that ortho-k results in a slower progression rate (less than -0.50 D/y). Additionally, the secondary null hypothesis for corneal curvature is that there is no significant change in pre- to post-treatment k values, suggesting temporary and reversible corneal reshaping effects of ortho-k after discontinuation [24,25]. Alternatively, the detection of a significant change in k values would indicate persistent corneal curvature alterations associated with ortho-k treatment. Testing these hypotheses will quantify ortho-k’s effectiveness in a real-world rural setting, assess its safety, and evaluate covariates such as age, sex, and baseline myopia to optimize myopia management strategies for underserved populations [26].

## Materials and methods

Study design

This retrospective chart review assessed the effectiveness of orthokeratology (ortho-k) in slowing myopia progression in adolescents. De-identified records from patients treated at a private optometry practice within a rural Appalachian community between 2000 and 2024 were analyzed.

Study population

Eligible participants were adolescents aged 8-16 years at the start of ortho-k treatment, with at least six months of treatment and documented baseline and follow-up refractive errors and corneal curvatures (k readings) between 21 and 90 days after treatment discontinuation [18]. Exclusion criteria included ocular conditions other than myopia (e.g., keratoconus), prior eye surgery, or incomplete records. Of 98 eyes (49 patients) initially identified, 90 eyes (45 patients) met criteria after excluding four patients due to incomplete records, ongoing treatment, insufficient treatment duration, or concurrent ocular conditions, consistent with sample sizes in similar studies [27]. Categorical patient data pertaining to the analyses conducted in this study are summarized in Table 1. Pre-treatment clinical data for study participants are summarized in Table 2.

**Table 1:** Summary of categorical data for the study participants OD: oculus dexter (right eye), OS: oculus sinister (left eye)

Participants	Number (N = 45)	%
Sex		
Male	25	56%
Female	20	44%
Eyes	Number (N = 90)	%
OD	45	50%
OS	45	50%

**Table 2:** Summary of pre-treatment clinical data for the study participants The BCVA for all patients in this study sample was 20/20. ^a^Refractive error in spherical equivalents, defined as sphere plus one-half the cylinder on cycloplegic refraction BCVA: best-corrected visual acuity, D: diopters, km: k mean, kd: k difference

	Mean	Standard deviation	Minimum	Maximum
Age, years	11.16	± 2.8	8	16
Treatment duration, months	53.82	± 32	6	147
Refractive error				
Sphere, D	-2.79	± 1.58	-0.75	-7.00
Cylinder, mm	-0.18	± 0.46	0.75	-2.00
Spherical equivalent, D^a^	-2.88	± 1.59	-0.75	-7.50
Corneal curvature				
Flatter meridian, D	42.89	± 1.46	39.95	46.88
Steeper meridian, D	43.78	± 1.59	40.86	47.83
km_i_, D	43.34	± 1.49	40.72	47.36
kd_i_, D	0.89	± 0.68	0.11	3.57

Data collection

Data were extracted from electronic and paper records at a private optometry practice in eastern Kentucky, including demographics (age, sex, and ethnicity), refractive error (diopters, D), corneal curvature (k1 and k2), and best-corrected visual acuity (BCVA). Records of ortho-k-treated patients from 2000 to 2024 were screened, and eligible data were entered into an Excel spreadsheet (Microsoft Corp., Redmond, WA) on a secure, on-site computer. Birth and visit dates were converted to age and treatment duration, with original dates discarded for de-identification. The de-identified dataset was transferred to a personal device for analysis.

Corneal curvature

Two metrics, k mean (km) and k difference (kd), were used to detect changes in corneal curvature using keratometry readings (k1 and k2) taken before and after ortho-k treatment. Changes in corneal curvature (Δkm and Δkd) were evaluated as secondary outcomes in this study.

Km represents the average curvature of the cornea in diopters and is calculated as the mean for k1 and k2. Change in k mean (Δkm) was calculated as the difference between pre-treatment (km_i_) and post-treatment (km_f_) mean curvatures (Δkm = km_f_ - km_i_), which was used to define the “flattened,” “non-flattened,” and “steepened” subgroups. The “flattened” subgroup included cases that demonstrated a decrease in mean corneal curvature after ortho-k treatment (Δkm > 0); the “non-flattened” subgroup included cases that did not show evidence of corneal flattening (Δkm ≤ 0); and the “steepened” subgroup included cases that demonstrated an increase in mean corneal curvature (Δkm < 0).

Kd is equal to the absolute difference between k values (kd = |k1 - k2|) and represents the measure of corneal astigmatism. Change in corneal astigmatism (Δkd) is the difference between pre- and post-treatment values, calculated as post-treatment minus pre-treatment (Δkd = kd_f_ - kd_i_).

Treatment details

Ortho-k lenses were Boston XO rigid gas-permeable lenses fitted using a Keratron™ Scout corneal topographer running factory software. Lenses were prescribed to patients with instructions to be worn nightly during sleep. Treatment duration was calculated using the clinic visit date at treatment start and the first post-treatment visit, ranging from 21 to 90 days of discontinuation, which allowed for the assumption of a sufficient post-treatment washout period [28,29].

Statistical analysis

Descriptive statistics were used to summarize demographic characteristics, refractive error, k readings, and adverse events, with means, standard deviations, and ranges reported for continuous variables and frequencies for categorical variables. The primary outcome (rate of myopia progression (rΔSE, D/y)) was derived from measured refractive error following conversion to spherical equivalents (SE), defined as sphere + one-half of the cylinder from cycloplegic autorefraction. Change in refractive error (ΔSE) was then calculated from pre- and post-treatment refractive values and divided per annualized duration of treatment in months (rΔSE = ΔSE / (# months of treatment / 12)), which allowed for direct comparison to the published norm of -0.50 D/y for untreated myopic adolescents using a one-sample t-test [6]. Paired t-tests were conducted to assess changes in refractive error, as Shapiro-Wilk results supported normal distributions (p = 0.116). Kolmogorov-Smirnov tests for normality confirmed the non-normal datasets, which were subsequently analyzed using Wilcoxon signed-rank tests to compare means. Secondary analyses examined the associations between progression rates, keratometry changes, and factors such as sex, age, treatment duration, and baseline myopia, using regression models where feasible, based on sample size [5]. Normal data was analyzed for Pearson correlation, while Spearman’s rho test was performed on data with non-normal distribution. To account for potential non-independence between the eyes of individual patients, concurrent patient-level analysis was performed using averaged eye data. When patient-level analysis was insufficient, generalized estimating equations (GEE) were used to model exchangeable correlation between eyes of the same patient. Data analytics were performed using IBM SPSS Statistics software (IBM Corp., Armonk, NY).

## Results

This retrospective chart review evaluated the efficacy of ortho-k in slowing myopia progression in 45 adolescents (90 eyes, 55.6% male patients, mean age: 11.1 ± 2.7 years) treated at a rural Appalachian optometry practice from 2000 to 2024. The primary outcome was the annualized rate of myopia progression in spherical equivalents compared to the published untreated progression rate of -0.50 D/y. Secondary outcomes included changes in corneal curvature, covariate effects (sex, age, treatment duration, and baseline myopia), and safety profile.

Myopia progression rates

**Figure 1:**
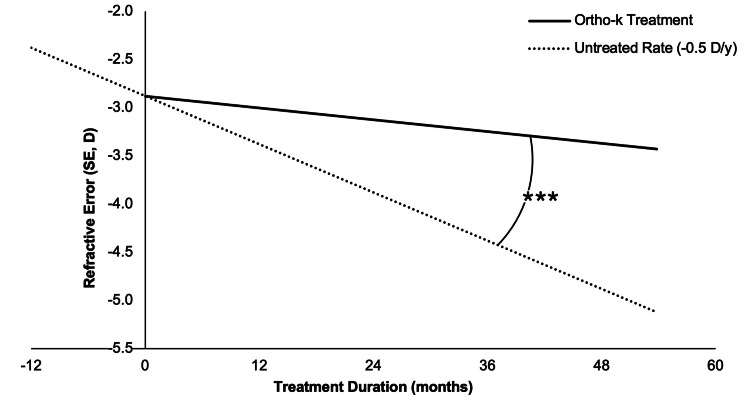
Myopia progression in adolescents treated with orthokeratology compared to the untreated progression rate of -0.50 D/y ΔSE are plotted as a function of the mean treatment duration (53.8 months) across all 45 study participants (N = 45), where rΔSE is illustrated by the slope of the connecting line. The untreated rate precedes the timing of treatment to depict the theoretical rate of progression prior to treatment. One-sample t-test, t(89) = 7.29, ***p < 0.001. Note: Asterisks denote the level of significance detected in evaluating differences between rΔSE, illustrated by the slopes of the corresponding lines. *p < 0.05, **p < 0.01, ***p < 0.001 ΔSE: mean pre- and post-treatment refractive errors in spherical equivalents, rΔSE: rate of myopia progression

**Figure 2:**
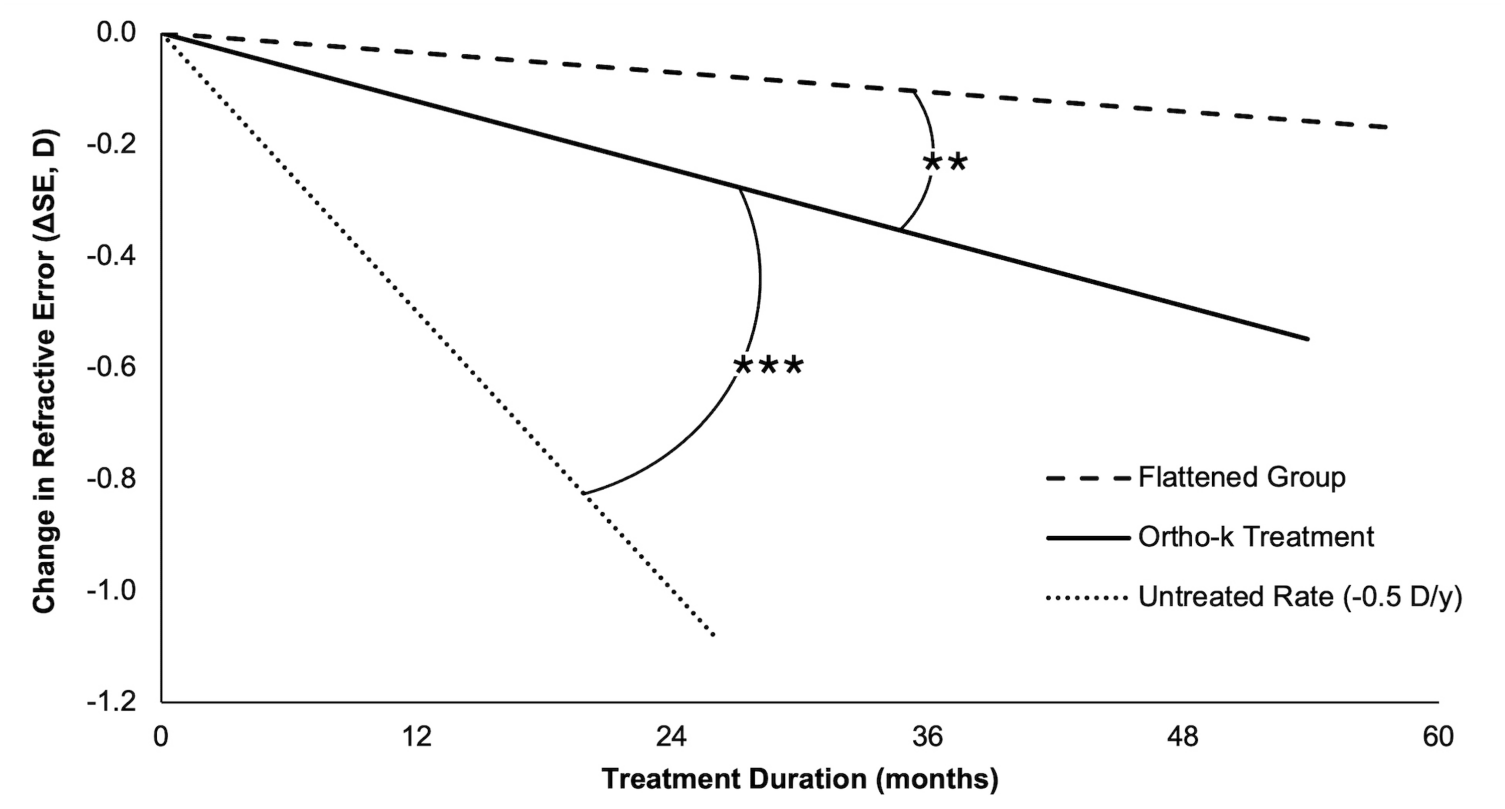
Myopia progression rates (rΔSE, D/y) in adolescents treated with ortho-k compared to the flattened group and the untreated progression rate of -0.50 D/y The graph shows mean post-treatment refractive errors relative to pre-treatment values in SE as a function of mean treatment duration (53.8 months). Progression rates are depicted as line slopes for the full ortho-k group (90 eyes, -0.227 D/y), the flattened group (11 eyes, -0.028 D/y, Δkm < 0), and a theoretical untreated group. The ortho-k treatment group demonstrated a significantly slower progression rate compared to the untreated rate (one-sample t-test, ***p < 0.001). The flattened subgroup demonstrated a significantly slower progression rate compared to the full ortho-k treatment group (Wilcoxon signed-rank test, **p = 0.003). *p < 0.05, **p < 0.01, ***p < 0.001 rΔSE: rate of myopia progression, SE: spherical equivalents, Δkm: change in k mean

The mean rate of myopia progression across 90 eyes was -0.227 ± 0.351 D/y, significantly slower than the untreated benchmark of -0.50 D/y (one-sample t-test, t(89) = 7.29, p < 0.001, Cohen’s d = 0.77) and represents a 55% reduction from the untreated rate (Figure 1) [6]. The large effect size and confidence interval (0.20-0.35 D/y) highlight a meaningful reduction in progression, supporting the efficacy of orthokeratology in slowing myopia progression in this sample. In the flattened subgroup (defined by post-treatment corneal flattening, Δkm < 0), the mean progression rate was -0.028 ± 0.134 D/y, which was significantly slower than the untreated rate of -0.50 D/y (Wilcoxon signed-rank test, Z = 2.936, p = 0.003) and demonstrates a 94% reduction (Figure 2). Patient-level analysis (mean = -0.20 ± 0.252 D/y) showed similar efficacy with a 60% reduction from the published untreated rate (t(44) = -6.270, p < 0.001, Cohen’s d = 0.935), indicating robust myopia control, particularly in the flattened subgroup [14].

Pre- and post-treatment k values

**Figure 3:**
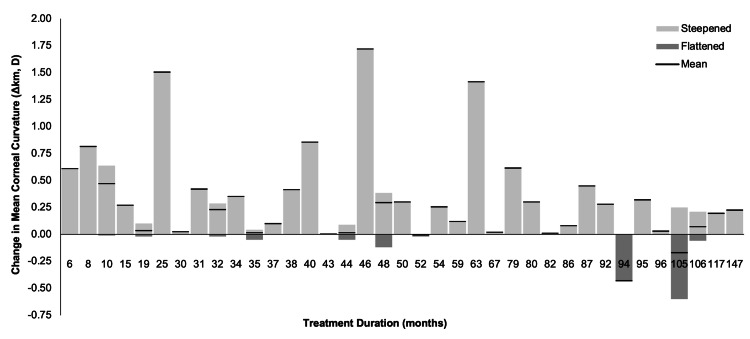
Distribution of the change in mean corneal curvature (Δkm, in diopters) for given treatment duration Lighter/upward bars indicate the sum of all corneal steepening (Δkm > 0) that was observed among the study participants for a given treatment duration. Darker/downward bars represent the sum of all corneal flattening (Δkm < 0) that was observed among the study participants for a given treatment duration. The black line overlying the bar for a given treatment duration represents the mean change that was observed among all study participants who were treated for that duration. Δkm: change in k mean

Wilcoxon signed-rank tests were used to compare pre- and post-treatment mean corneal curvatures (Δkm). In the full study sample of patients treated with ortho-k (90 eyes), post-treatment mean curvature was significantly greater than pre-treatment mean curvature (Z = -6.776, p < 0.001), with a mean Δkm of 0.327 ± 0.445 D. Meaning that, on average, ortho-k patients included in this study demonstrated slightly steeper/more curved corneas when evaluated following ortho-k treatment compared to their pre-treatment baselines. Also, in the full study sample, Δkm showed no correlation with treatment duration, which can be inferred by the lack of any appreciable trend in Figure 3, and lends support to the assumption of an adequate post-treatment washout period prior to clinical evaluation. No significant change was observed in corneal astigmatism (Δkd = kd_f_ - kd_i_) (Z = -0.344, p = 0.731), with a mean difference of 0.023 ± 0.418 D. Patient-level analysis confirmed slight steepening in Δkm (Z = -5.147, p < 0.001) and no change in Δkd (Z = -0.327, p = 0.744). These results suggest that ortho-k promotes corneal flattening while maintaining astigmatism stability [15].

Correlation between k values and myopia progression

**Figure 4:**
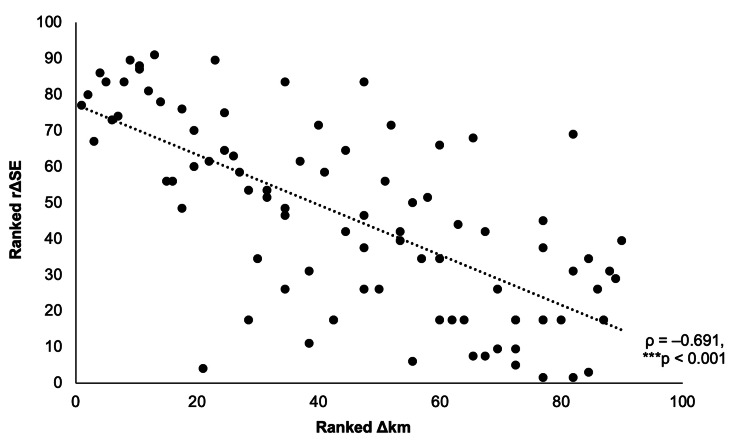
Scatterplot showing the relationship between change in mean corneal curvature (Δkm, in diopters) and myopia progression rate (rΔSE, D/y) in orthokeratology-treated adolescents Points plotted for individual eyes (N = 90) represented in the study, with the dashed trendline indicating a strong negative correlation (Spearman’s rank correlations: ρ = -0.691, ***p < 0.001), linking greater flattening to slower progression. Δkm: change in k mean, rΔSE: rate of myopia progression

Spearman’s rank correlations were used to assess the relationships between k values and myopia progression. At the eye level, myopia progression (rΔSE) was strongly negatively correlated with change in mean corneal curvature (Δkm) (ρ = -0.691, p < 0.001), indicating that greater corneal flattening slowed progression (Figure 4). No correlation was found with change in corneal astigmatism (Δkd) (ρ = 0.073, p = 0.495). Pearson’s correlation confirmed a strong negative association between ΔSE and Δkm (r = -0.699, p < 0.001). Patient-level analysis showed a similar negative correlation between rΔSE and Δkm (ρ = -0.687, p < 0.001), but not with Δkd (ρ = 0.160, p = 0.293). These findings support corneal flattening as a key mechanism for myopia control [13].

Sex-based covariate evaluation

A generalized estimating equation (GEE) model with an unstructured correlation structure was used to assess sex differences in an adjusted progression rate (r’ΔSE = rΔSE + 0.5). The model included Sex, corneal flattening (Δkm), and their interaction (Sex × Δkm) as predictors. In the full sample (90 eyes, 45 patients, 55.6% male patients), no significant sex effect was observed (Wald χ² = 0.012, p = 0.911), nor was the Sex × Δkm interaction significant (Wald χ² = 0.016, p = 0.899). However, Δkm significantly predicted r’ΔSE (Wald χ² = 4.811, p = 0.028, B = 0.787, SE = 0.296), further indicating that greater corneal flattening was associated with slower myopia progression.

**Figure 5:**
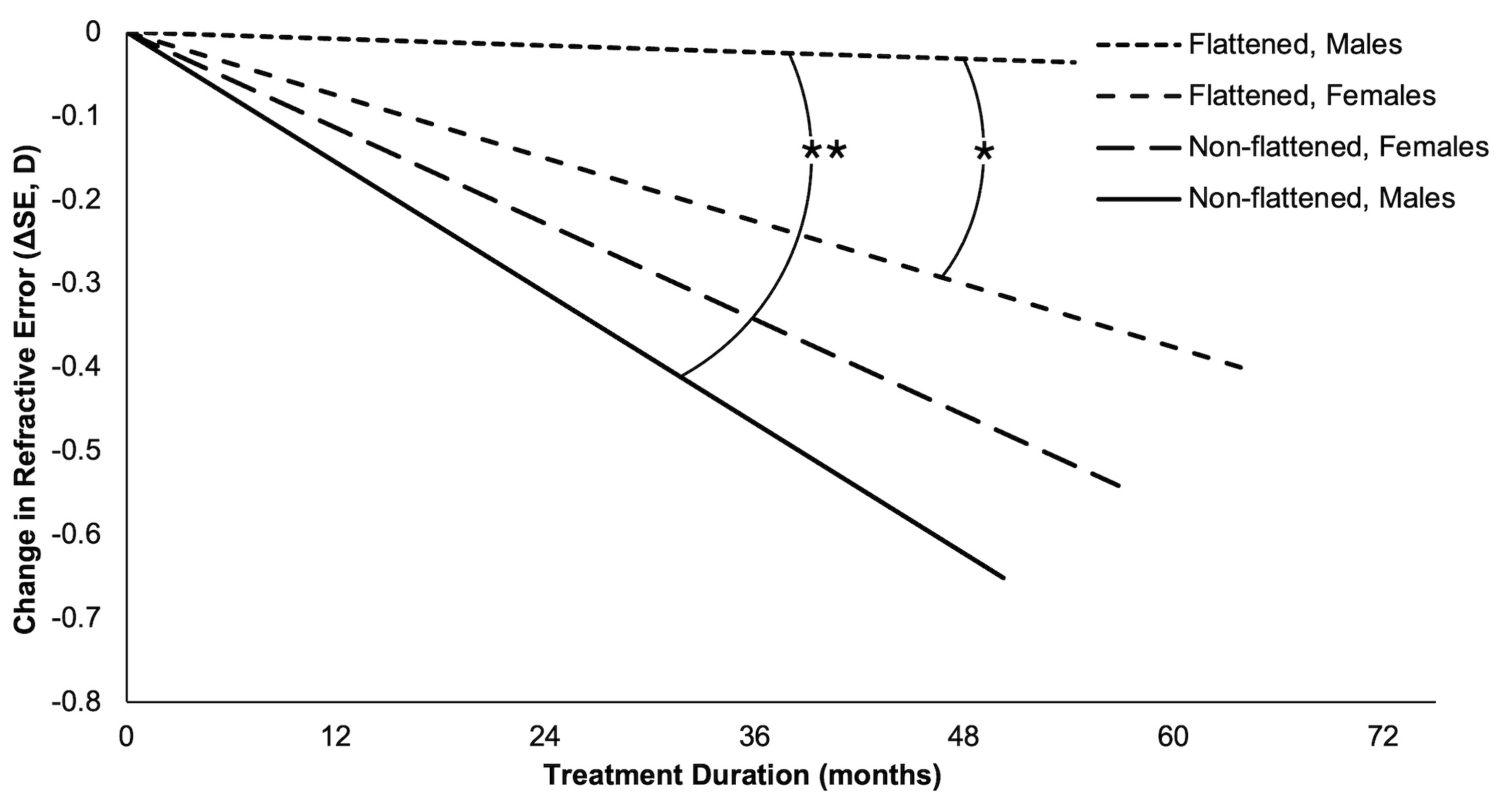
Myopia progression rates (rΔSE, D/y) for male and female patients in flattened (Δkm < 0) and non-flattened (Δkm ≥ 0) subgroups, plotted by mean treatment duration Male patients in the flattened subgroup (n = 7 eyes, 0.025 ± 0.110 D/y) showed significantly slower progression than non-flattened male patients (n = 43 eyes, -0.26 ± 0.33 D/y, Wilcoxon signed-rank test, **p = 0.0048) and flattened female patients (n = 4 eyes, -0.122 ± 0.130 D/y, generalized estimating equation, *p = 0.014). No significant differences were observed between sexes in the non-flattened subgroup or between female subgroups. rΔSE: rate of myopia progression, Δkm: change in k mean

In the flattened subgroup (11 eyes, 10 patients, mean Δkm = -0.165 ± 0.222 D), a GEE model showed a significant sex effect (Wald χ² = 6.012, p = 0.014) and a significant Sex × Δkm interaction (Wald χ² = 6.629, p = 0.010). Male patients (seven eyes) had a higher r’ΔSE (0.525 ± 0.110 D/y) than female patients (four eyes, 0.364 ± 0.130 D/y) (Cohen’s d = 1.20), which was supported by the parameter estimate for female patients (B = -0.243, SE = 0.099, p = 0.014) that indicated faster myopia progression relative to male patients. The Sex × Δkm interaction (B = -0.535, SE = 0.208, p = 0.010) suggests that corneal flattening enhanced efficacy more in male patients than in female patients (B for *Δkm* = 0.254, p = 0.002) [30]. This effect is illustrated as a function of treatment duration in Figure 5. One-sample Wilcoxon signed-rank tests indicated that r’ΔSE for male patients significantly differed from zero (Z = 2.371, p = 0.018), unlike female patients (Z = 1.826, p = 0.068), further supporting greater treatment efficacy in male patients.

Nonsignificant covariates: age, treatment duration, and baseline myopia

**Figure 6:**
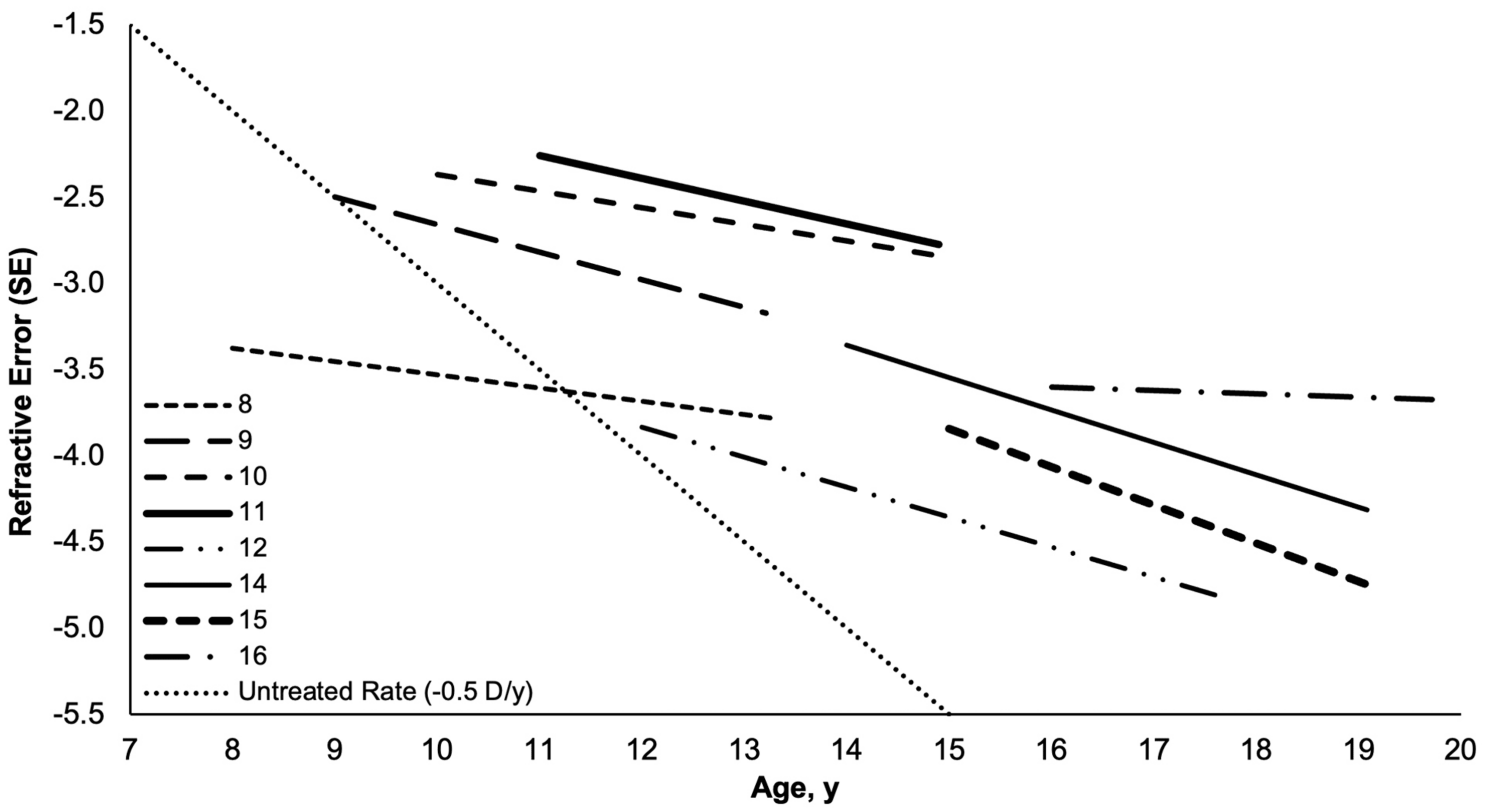
Mean change in refractive error (SE) for each age group (years) at treatment start, plotted to show the progression of age during treatment for the mean treatment duration of the given age group The slopes of plotted lines represent myopia progression rates (rΔSE, D/y) per age group, shown with transversal trendline representing the untreated rate (-0.50 D/y) for comparison. Data from one 13-year-old with positive myopia correction were excluded to avoid skewing trends. rΔSE: rate of myopia progression

In the full sample, Age showed a weak negative correlation with progression rate (rΔSE, ρ = -0.15, p = 0.162), suggesting that younger patients had slightly faster progression (Figure 6) [5]. However, age, treatment duration, and baseline myopia showed no significant effects on myopia progression or corneal curvature (all p > 0.05), indicating consistent ortho-k efficacy across these factors.

Safety profile

No adverse events (e.g., corneal infections and abrasions) were reported across 90 eyes, consistent with the safety profile of ortho-k [18].

## Discussion

Initial findings and significance

This retrospective chart review of 45 adolescents (90 eyes) treated with orthokeratology (ortho-k) in a rural Appalachian community demonstrates that ortho-k significantly slows myopia progression compared to the published untreated progression rate of -0.50 D/y [6]. The mean annualized progression rate of -0.227 ± 0.351 D/y was significantly slower than this benchmark (p < 0.001). These findings align with those from previous randomized trials such as the Retardation of Myopia in Orthokeratology (ROMIO) study, which reported a 43% reduction in axial length elongation with ortho-k [14], and the meta-analysis by Si et al. that found that ortho-k reduces myopia progression by approximately 45% [6], consistent with the 55% reduction observed in our study. Notably, the subgroup with corneal flattening (Δkm < 0) exhibited a remarkably low progression rate of -0.028 ± 0.134 D/y (94% reduction from baseline; 88% reduction from the full study sample), highlighting the pivotal role of corneal reshaping in myopia control.

The identification and analysis of the flattened subgroup (Δkm < 0) in this study highlight a critical and underexplored aspect of orthokeratology’s impact on myopia control. During adolescence, corneal steepening typically accompanies eye elongation and enlargement, a natural part of ocular development. Observing the opposite effect (corneal flattening) in a subset of ortho-k-treated adolescents strongly suggests a direct treatment effect. This makes the flattened subgroup particularly significant for isolating ortho-k’s role in altering corneal curvature. While ortho-k likely influenced cases with unchanged (Δkm = 0) or minimally altered curvature, attributing such outcomes solely to treatment is less reliable. Thus, focusing on cases with reduced corneal curvature enhances confidence in linking ortho-k to slowed myopia progression.

An intriguing finding emerged in the flattened subgroup, where male patients exhibited slower progression than female patients (p = 0.014), despite no significant sex differences in the full sample (p = 0.911). This contrasts with Jones-Jordan et al., who reported faster progression in female patients in a non-ortho-k cohort [5]. However, our study’s higher baseline myopia in male patients (-3.27 D versus -2.39 D in female patients, p = 0.009) may have influenced treatment response, despite only the slight correlation between baseline myopia and progressive rate during treatment that was detected in this study. The small female sample in the flattened subgroup (n = 4 eyes) limits definitive conclusions; however, this sex-specific trend warrants further exploration, particularly as baseline myopia severity may modulate ortho-k outcomes [11].

A notable finding from this study was the minimal influence of treatment duration on myopia progression rates. Given that nightly ortho-k lens wear modifies corneal architecture to slow myopia progression, one might expect dynamic effects tied to treatment duration, such as an initial pronounced effect that diminishes over time, a delayed response due to gradual corneal reshaping, or fluctuations linked to hormonal changes during puberty. Contrary to these expectations, the results of the current study revealed remarkable consistency, with ortho-k patients maintaining a significantly reduced progression rate independent of treatment duration, showing negligible variance. This study’s mean treatment duration of 53.8 months exceeds that of many trials, suggesting sustained long-term efficacy, as supported by Jakobsen et al., who demonstrated continued myopia control over three years with ortho-k [31]. This prolonged effect is particularly relevant in adolescents, where myopia progression typically peaks during puberty [1]. The stability in treatment demonstrated by the results of this study further supports the strategic use of ortho-k in early adolescence to minimize the severity of refractive errors, often as a preparatory step for patients planning laser-assisted in situ keratomileusis (LASIK) correction upon reaching eligibility at age 18 [32].

Age and baseline myopia severity had minimal impact on myopia progression rates in this study, consistent with Santodomingo-Rubido et al., who reported no significant influence of these factors on ortho-k efficacy [27]. Although younger patients exhibited slightly faster progression, this effect was not statistically significant and did not diminish the overall slowed progression rate attributed to ortho-k treatment (-0.227 ± 0.351 D/y). Similarly, baseline myopia severity had no significant impact on treatment outcomes. While selection bias might be present due to practice providers favoring ortho-k trials for patients with myopia less severe than -5.00 D, it is noteworthy that the few patients with higher baseline myopia, including one at -7.00 D, demonstrated treatment responses comparable to the broader study sample, underscoring ortho-k’s efficacy across a range of myopia severities.

Practical implications

From a clinical perspective, the study’s findings underscore the potential for ortho-k to be a cornerstone of myopia management in rural optometric practices. The significant reduction in progression rate (55% overall and 94% in the flattened subgroup) and the absence of adverse events highlight ortho-k’s feasibility as a safe, effective intervention in settings with limited access to specialized care. For rural practitioners, ortho-k offers a non-invasive, reversible option that can be tailored to adolescent patients, potentially reducing the need for frequent follow-ups compared to other modalities, such as atropine. The increasing prevalence of myopia and its potential to result in significant vision impairment emphasize the urgent need for effective interventions [33]. In this context, the ability of ortho-k to slow myopia progression by 55% overall and up to 94% in the flattened subgroup represents a clinically meaningful strategy for managing this growing issue among adolescents at risk. Training programs for eye care practitioners in underserved areas could prioritize ortho-k fitting and management to expand its adoption, aligning with the need for scalable solutions in regions with healthcare disparities.

Another critical aspect of this study is its focus on post-treatment effects within 90 days of discontinuing ortho-k, providing insight into the persistence of treatment benefits. The observed corneal steepening post-treatment (Δkm = 0.327 ± 0.445 D) in the full sample suggests a partial return to baseline corneal curvature. However, the flattened subgroup’s minimal progression rate (-0.028 ± 0.134 D/y) indicates that corneal flattening may confer lasting myopia control benefits. This finding is consistent with Hiraoka et al., who reported residual myopia control effects after ortho-k cessation [16], potentially due to sustained changes in peripheral refraction [34]. Future studies should explore the duration and mechanisms of these residual effects to optimize treatment protocols and discontinuation strategies.

The absence of significant covariate effects (age, treatment duration, and baseline myopia) in the full sample underscores ortho-k’s broad applicability across adolescent populations. However, the sex-specific findings in the flattened subgroup suggest that individual physiological factors, such as corneal biomechanics or hormonal influences, may modulate treatment response. For instance, differences in corneal thickness or elasticity between male and female patients could influence the degree of corneal flattening achieved, as suggested by preliminary studies on corneal hysteresis [34-36]. Incorporating such biomechanical assessments in future research could refine patient selection and enhance treatment outcomes.

The safety profile of ortho-k in this study was exemplary, with no adverse events reported across 90 eyes, corroborating the findings of Cho and Tan, who noted low complication rates with proper ortho-k use [18]. The rural Appalachian setting may have contributed to this outcome, as limited access to digital devices and reduced near-work (factors linked to slower baseline progression) could enhance the relative efficacy of ortho-k [9,37].

Limitations

Several limitations temper conclusions drawn from the results of this study. The absence of axial length measurements, a gold standard in myopia control studies such as the Correction of Myopia Evaluation Trial [38,39], limits mechanistic insights; however, changes in refractive error provide a robust proxy. Imprecision of the 21- to 90-day washout period, between treatment discontinuation and the collection of post-treatment clinical data, compromises the ability to confirm stability of clinical outcomes. The retrospective design and single-center setting introduce potential selection bias, although the rural Appalachian cohort offers unique real-world insights into ortho-k’s practical application [9]. The small sample size of the study group, especially female patients in the flattened subgroup (four eyes), restricts the generalizability of the results, particularly those that are sex-specific. Additionally, the non-normal distribution of some variables (e.g., rΔSE, overall: skewness = -2.37; flattened: female patients skewness = -1.99; Δkm, p = 0.003; Δkd, p = 0.033) necessitated non-parametric analyses, potentially reducing statistical power.

## Conclusions

This study demonstrates that ortho-k effectively slows myopia progression in adolescents, with pronounced benefits in those exhibiting corneal flattening. The strong safety profile and sustained efficacy over an average of 53.8 months support ortho-k as a viable myopia control strategy, particularly in rural settings where environmental factors may amplify its effects. These findings underscore the importance of corneal reshaping as a central mechanism of action and highlight the need for further research into individual response variations, such as sex differences, to optimize the application of ortho-k in clinical practice.
